# Morphological and molecular characterization of *Isospora elliotae* n. sp. (Apicomplexa: Eimeriidae) from the Australian magpie *Gymnorhina tibicen* (Latham, 1801) (Passeriformes: Artamidae) in Western Australia

**DOI:** 10.1002/ece3.10505

**Published:** 2023-09-05

**Authors:** Rongchang Yang, Siobhon Egan, Huimin Gao, Belinda Brice, Bruno P. Berto

**Affiliations:** ^1^ Australian National Phenome Centre, Health Futures Institute Murdoch University Perth Western Australia Australia; ^2^ Institute of Cash Crops Hebei Academiy of Agriculture and Forestry Sciences Shijiazhuang China; ^3^ Kanyana Wildlife Rehabilitation Centre Lesmurdie Western Australia Australia; ^4^ Departamento de Biologia Animal, Instituto de Ciências Biológicas e da Saúde Universidade Federal Rural do Rio de Janeiro Seropédica Brazil

**Keywords:** 18S rRNA gene, 28S rRNA gene, Australian magpie, coccidia, COI gene, *Isospora*

## Abstract

A new coccidian species, *Isospora elliotae* n. sp., from the Australian magpie *Gymnorhina tibicen* (Latham, 1801) in Western Australia, is described and characterized morphologically and molecularly. Microscopic analysis of a faecal sample identified subspheroidal oocysts (*n* = 20), 20–22 × 18–20 (20.7 × 18.7); length/width (L/W) ratio 1.05–1.14 (1.10). Wall bi‐layered, 1.0–1.3 (1.2) thick, outer layer smooth, *c*. 2/3 of total thickness. Micropyle and oocyst residuum absent, but usually two polar granules are present. Sporocysts (*n* = 28) ovoidal, 12–13 × 9–11 (12.6 × 9.7); L/W ratio 1.22–1.35 (1.30). Stieda body present, flattened to half‐moon‐shaped, *c*. 0.5 deep × 2.0 wide; sub‐Stieda indistinct or barely discernible, *c*. 1.0 deep × 2.5 wide; para‐Stieda body absent; sporocyst residuum present, composed of granules dispersed among the sporozoites. Sporozoites vermiform, with anterior and posterior refractile bodies and nucleus. Segments of three gene *loci* (18S rRNA, 28S rRNA and COI) were sequenced and *I*. *elliotae* n. sp. exhibited 99.8% genetic similarity to *Isospora* sp. MAH‐2013a (KF648870) followed by 99.7% genetic similarity to *Isospora neochmiae* (Yang, Brice & Ryan, 2016) (KT224380) at the 18S rRNA gene locus. It shared 97.0% genetic similarity with an unnamed *Isospora* sp. (AY283852) at the 28S rRNA gene locus and it also shared the highest genetic similarity of 99.8% with the unnamed *Isospora* sp. from an American crow (OL999120) at the COI gene locus. Based on morphological and molecular data, this isolate is a new species named as *I*. *elliotae* n. sp.

## Graphical abstract

Description of *I*. *elloitae* n. sp. in an Australian magpie. Morphology: unique. Genetic: 99.8% similar to *Isospora* sp. MAH‐2013a (KF648870) from Canada at 18S locus.

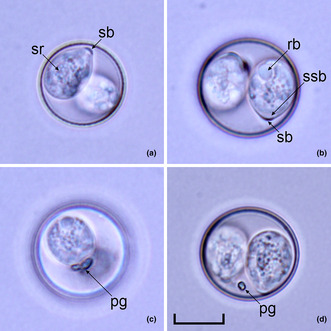

## 1 INTRODUCTION

Coccidia are microscopic single‐celled protozoan parasites that are members of the Apicomplexa phylum and family Eimeriidae. These obligate intracellular parasites are found worldwide and tend to be mostly host specific (Greiner, 2008), infecting both invertebrates and vertebrates (Fayer, 1980). Birds and reptiles are commonly infected with coccidia. Faeces of captive and wild avian species often contain coccidian oocysts and multiple species of the unicellular parasite may infect the same host simultaneously. Most coccidian infections in wild birds are asymptomatic, however young immunocompromised birds and stressed adults are most likely to exhibit clinical signs of coccidiosis. The coccidia belonging to the genus *Isospora* Schneider, 1881, are the most common coccidians to infect the passerine birds (Duszynski & Wilber, 1997), of which the Australian magpie (*Gymnorhina tibicen* Latham, 1801) is one. The Australian magpie is a member of the Artamidae family and is found throughout the Australian continent, except in the driest of the desert areas (Pizzey & Knight, 2007). More than 500 species of *Isospora* have been reported from passerine birds (Berto et al., 2011; Greiner, 2008) and numerous *Isospora* spp. have now been characterized (Schoener et al., 2013; Schrenzel et al., 2005). To date, only one species of *Isospora* has been characterized from the Artamidae family worldwide, namely *I*. *streperae* from a grey currawong *Strepera versicolor* (Latham, 1801) in Western Australia, which was characterized by Yang, Brice, Al Habsi et al. (2015).

Faecal screening for intestinal parasites is routinely performed on all Australian magpies admitted to the Kanyana Wildlife Rehabilitation Centre (KWRC), Perth, Western Australia. Many young Australian magpies admitted to the KWRC are infected with coccidia. Other intestinal parasites identified in the faeces from Australian magpies at KWRC include nematodes such as *Capillaria* spp., cestode ova and *Trichomonas* spp. Fledgling and juvenile Australian magpies are also frequently infected with throatworm, (*Cheilospirura gymnorhinis* de Chaneet & Robertson, 1983) and many Australian magpies admitted to the KWRC have concurrent throatworm and coccidian infections. Australian magpies that are clinically ill with coccidiosis often exhibit clinical signs of discomfort and pain. Other clinical signs include lethargy, fluffed up feathers, weight loss due to inappetence, dehydration, a dirty vent, smelly faeces and diarrhoea. Subclinical infections may also result in sudden death as reported by Ball et al. (1998).

The taxonomy of the *Isospora* genus has been controversial due to the paraphylogeny of the genus and differences in morphology among the oocysts (Barta et al., 2005). Since 2013, we have had the opportunity to contribute to the knowledge of coccidian species infecting passerine birds of Western Australia using both traditional morphological and modern multiple‐loci molecular approaches. In this study, we characterize a new *Isospora* species from the Australian magpie and propose the species name *I*. *elliotae* n. sp.

## 2 MATERIALS AND METHODS

### 2.1 Sample collection

A wild juvenile Australian magpie, (*G*. *t*. *dorsalis*) was admitted to KWRC in July 2014 after it was found by a member of the public. Veterinary examination and radiological assessment revealed a poor body condition score, haemorrhage mid ulna with a non‐displaced fracture of the mid right ulna. A further X‐ray was performed a few days later as the bird developed severe dyspnoea. A tumour was found to be present in the thoracic inlet. Severe air sac congestion was also observed so the bird was humanely euthanized.

Faecal samples were collected on admission as per the KWRC Australian magpie admission protocol. All procedures were also approved and monitored by Murdoch University Animal Ethics Committee (approval number R2352/17).

### 2.2 Morphological analysis

Microscopic examination of wet mounts as well as faecal float analysis was performed. Faecal flotation was done using a saturated sodium chloride and 50% sucrose (w/v) solution. Microscopy revealed large numbers of unsporulated coccidian oocysts. A portion of the faecal sample was placed in a sterile sample container, labelled and a 2% (w/v) potassium dichromate solution (K_2_Cr_2_O_7_) was added. The sample was thoroughly emulsified and stored at 4°C until transport to Murdoch University (within 24 h), for further investigation.

On arrival at the Murdoch university laboratory, a portion of the emulsified faecal sample solution was poured into the base of a Petri dish, to a depth of less than a cm and stored in a cupboard in the dark, at room temperature (20–22°C). Sporulated oocysts were observed using an Olympus DP71 digital microimaging camera. Images were taken using Nomarski contrast with a 100X oil immersion objective.

### 2.3 DNA extraction, PCR amplification, sequencing and phylogenetic analysis

Total DNA was extracted from 200 mg of each faecal sample using a Power Soil DNA Kit (MolBio) with some modifications. Briefly, samples were subjected to four cycles of freeze/thaw by liquid nitrogen and boiling water to ensure efficient lysis of oocysts before being processed using the manufacturer's protocol. A negative control (no faecal sample) was included.

Generic apicomplexan primers (CRYPTOF 5′‐AAC CTG GTT GAT CCT GCC AGT and CRYPTOR 5′‐GCT TGA TCC TTC TGC AGG TTC ACC TAC) were used to amplify the almost full length 18S rRNA gene as described by Eberhard et al. (1999). The expected PCR product was ~1584 bp. The PCR reaction contained 2.5 μL of 10 × Kapa PCR buffer, 3 μL of 25 mM MgCl_2_, 1.5 μL of 10 nM dNTP's, 10 pM of each primer, 1 unit of KapaTaq (Geneworks), 1 μL of DNA (~50 ng) and 14.9 μL of H_2_O. PCR cycling conditions were 1 cycle of 94°C for 3 min, followed by 45 cycles of 94°C for 30 s, 55°C for 30 s and 72°C for 2 min and a final extension of 72°C for 5 min.

The PCR for the 28S rRNA locus was carried out using a nested PCR with the external primers: 28SExF: 5′‐TAC CCG CTG AAC TTA AGC and 28SExR: 5′‐ CMA CCA AGA TCT GCA CTA G as previously described (Schrenzel et al., 2005), which produced a PCR product size of ~1495 bp. The internal primers (28InF: 5′‐ACT ATG TTC CCT AGT AAC G and 28SInR 5′‐AAC GCT TCG CCA CGA TCC) were as previously reported (Yang et al., 2014) and produced an amplicon size of 1420 bp. The PCR reaction contained 2.5 μL of 10 × Kapa PCR buffer, 2 μL of 25 mM MgCl_2_, 1 μL of 10 mM dNTP's, 10 pM of each primer, 1 unit of KapaTaq (Geneworks), 1 μL of DNA (~50 ng) and 16.9 μL of H_2_O. Both primary and secondary PCR's were conducted using the same cycling conditions; 1 cycle of 94°C for 3 min, followed by 35 cycles of 94°C for 30 s, 60°C for 30 s and 72°C for 90 s and a final extension of 72°C for 5 min.

A partial mitochondrial cytochrome oxidase gene (COI) gene sequence (723 bp) was amplified using a nested PCR with the following primers COIF1 (Ogedengbe et al., 2011) and COXR1 (Dolnik et al., 2009) for the external reaction and COIF2 (Yang, Brice, et al., 2013) and COXR2 (Dolnik et al., 2009), for the internal reaction.

The amplicons from the second round PCRs were gel purified using an in‐house filter tip method as previously described (Yang, Murphy, et al., 2013). All the PCR products were sequenced using forward and reverse primers in duplicate using amplicons from different PCR runs. An ABI PrismTM Terminator V3.1 Cycle Sequencing kit (Applied Biosystems) was used for Sanger sequencing according to the manufacturer's instructions. The results of the sequencing reactions were analysed and edited using Finch TV® v1.4.0. (http://www.geospiza.com/Products/finchtv.shtml). Phylogenetic trees were constructed for *Isospora* spp. at the 18S, 28S and COI loci with additional isolates from GenBank. Parsimony analyses were conducted using MEGA‐X (Molecular Evolutionary Genetics Analysis software (Neighbor‐joining (NJ) and maximum likelihood (ML) analyses were conducted using Tamura‐Nei based on the most appropriate model selection (TN93 + G for 18S rRNA; TN93 + G + I for 28S rRNA and GRT + G for COI gene, respectively) using ModelTest in MEGA‐X. Bootstrap analyses were conducted using 1000 replicates to assess the reliability of inferred tree topologies.

## 3 RESULTS

### 3.1 Morphological analysis

#### 3.1.1 Species description

**FIGURE 1:**
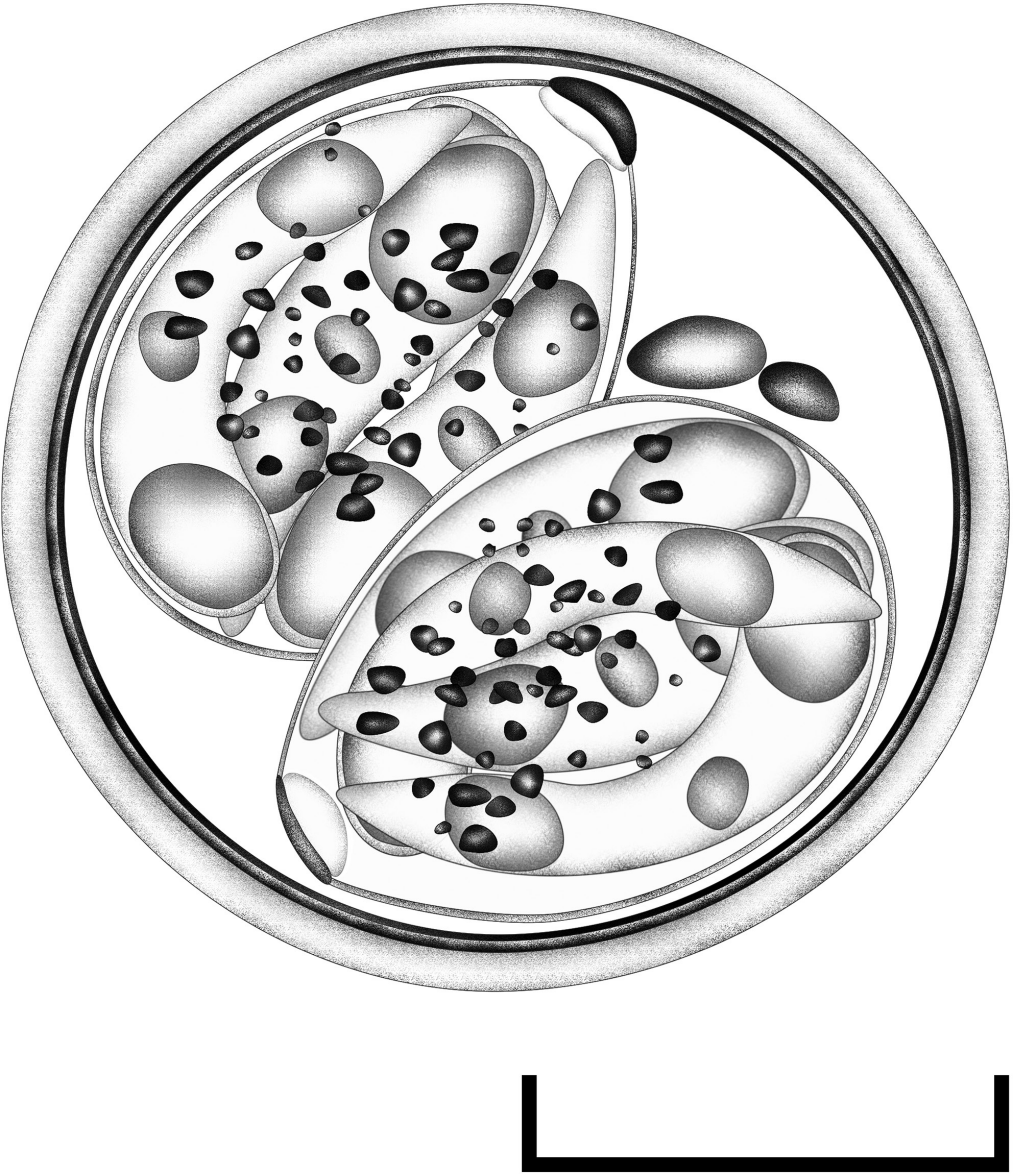
Composite line drawing of *Isospora elliotae* n. sp. sporulated oocyst. Scale bar = 20 μm.

**FIGURE 2:**
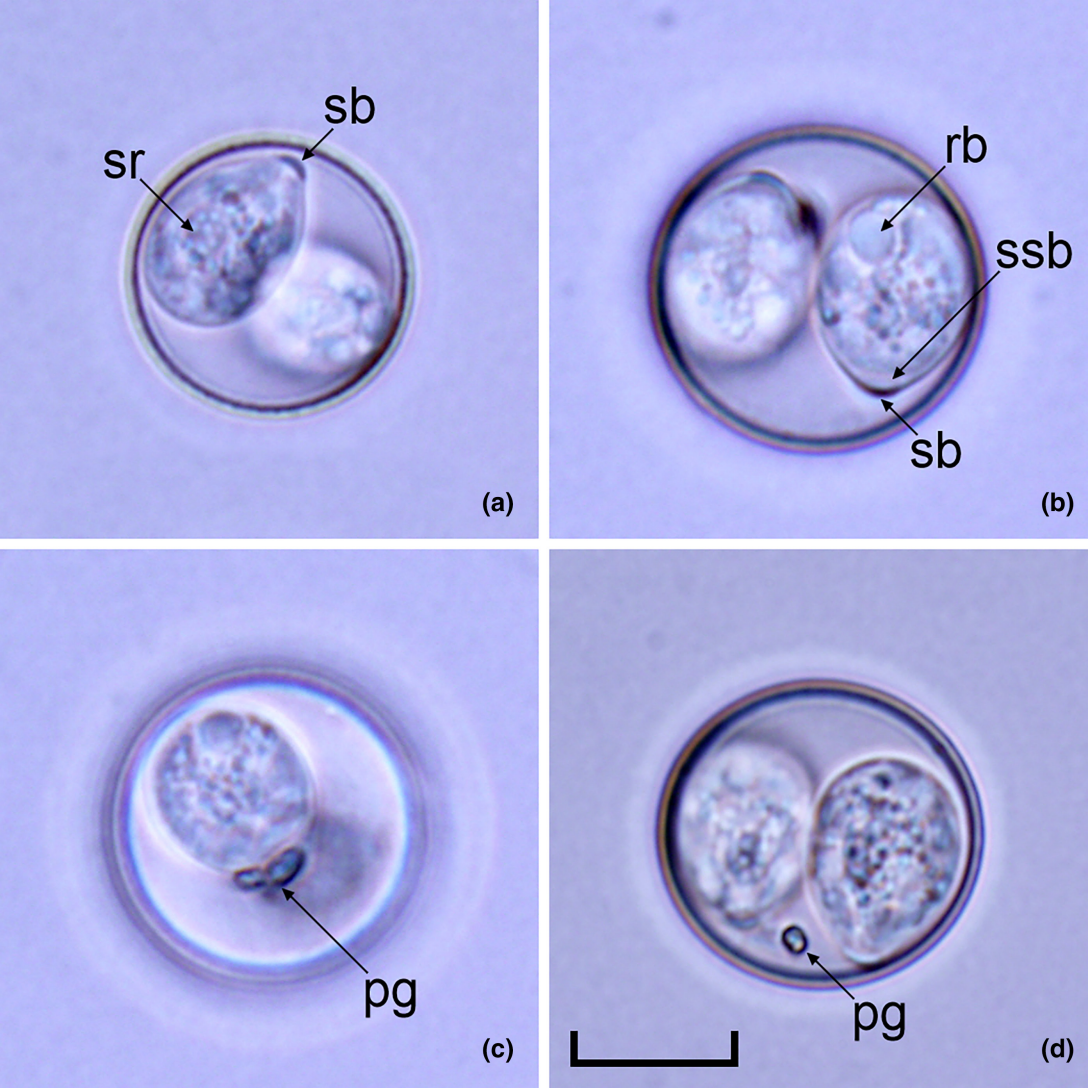
Nomarski interference‐contrast photomicrographs of *Isospora elliotae* n. sp. Scale bar = 20 μm.

Oocysts (*n* = 20) subspheroidal, 20–22 × 18–20 (20.7 × 18.7); length/width (L/W) ratio 1.05–1.14 (1.10). Wall bi‐layered, 1.0–1.3 (1.2) thick, outer layer smooth, *c*. 2/3 of total thickness. Micropyle and oocyst residuum absent, but usually two polar granules are present. Sporocysts (*n* = 28) ovoidal, 12–13 × 9–11 (12.6 × 9.7); L/W ratio 1.22–1.35 (1.30). Stieda body present, flattened to half‐moon‐shaped, *c*.0.5 deep × 2.0 wide; sub‐Stieda indistinct or barely discernible, *c*. 1.0 deep × 2.5 wide; para‐Stieda body absent; sporocyst residuum present, composed of granules dispersed among the sporozoites. Sporozoites vermiform, with anterior and posterior refractile bodies and nucleus (Figures 1 and 2).

#### 3.1.2 Host: Australian magpie (*Gymnorhina tibicen*)

Locality: Marmion, Perth, Western Australia (‐31.844° S, 115.751° E).

Other hosts: Unknown.

Prepatent period: Unknown.

Patent period: Unknown.

Site of infection: Unknown, oocysts collected from faeces.

Sporulation time: 48–72 h.

Material deposited: Oocysts in 10% formalin and oocyst photosyntypes were deposited in the Western Australian Museum under the reference number WAM Z68804.

Etymology: This species is named *Isospora elliotae* n. sp. after Aileen Elliot, a parasitologist from Murdoch University, Australia. Aileen Elliot has made a tremendous contribution to our taxonomic understanding of parasites of Australian wildlife, through her excellent skills as a parasitologist and her willingness to help generations of researchers and students.

### 3.2 Phylogenetic analysis

#### 3.2.1 18S rRNA

**FIGURE 3:**
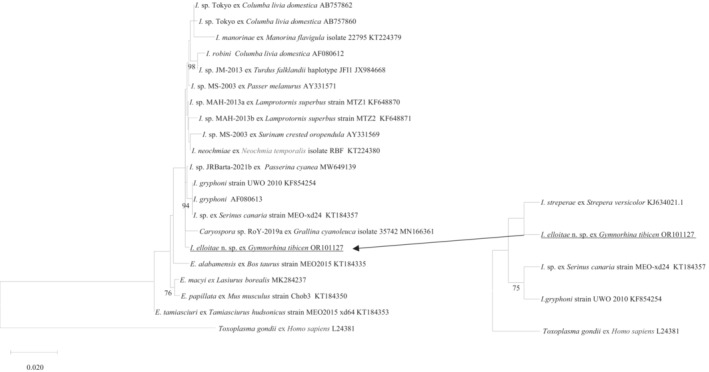
Evolutionary relationships of *Isospora elliotae* n. sp. inferred by distance analysis of 18S rRNA sequences. Percentage support (>70%) from 1000 pseudoreplicates from ML analysis.

Five identical 1302 bp 18S rRNA sequences were obtained from the faecal samples of *I*. *elliotae* n. sp., which was grouped with three *Isospora* spp. from Canada, one unnamed species (strain MEO‐xd24, KT184357) and two *I*. *gryphoni* (KF854254 and AF080613) sequences. Due to different primers being used for the amplification of the 18S rRNA of *I*. *streperae* and for *I*. *elliotae* n. sp., a sub‐tree with a 750 bp common sequence were generated (Figure 3). Unsurprisingly, *I*. *elliotae* n. sp. and *I*. *streperae* sat in the same clade.

The pair‐wise distance analysis revealed that *I*. *elliotae* n. sp. shared the highest genetic similarity of 99.8% with *Isospora* sp. MAH‐2013a (KF648870) identified from a superb glossy starling (*Lamprotornis superbus* Ruppell, 1845) in Canada, followed by 99.7% genetic similarity with *I*. *neochmiae* (KT224380) from a captive‐bred red‐browed finch (*Neochmia temporalis* Latham, 1801) in Western Australia (Yang, Brice, & Ryan, 2016) and it shared the same genetic similarity with another three *Isospora* spp. identified from birds in Canada: *Isospora* sp. strain MEO‐xd24 (KT184357) from an Atlantic canary (*Serinus canaria* Linnaeus, 1758), *I*. *gryphoni* (KF854254) and *Isospora* sp. (MW649139) from an indigo bunting (*Passerina cyanea* Linnaeus, 1766). In addition, *I*. *elliotae* n. sp. also showed 99.6% genetic similarity with another *I*. *gryphoni* (AF080613), which was also identified from Canada.

#### 3.2.2 28S rRNA

**FIGURE 4:**
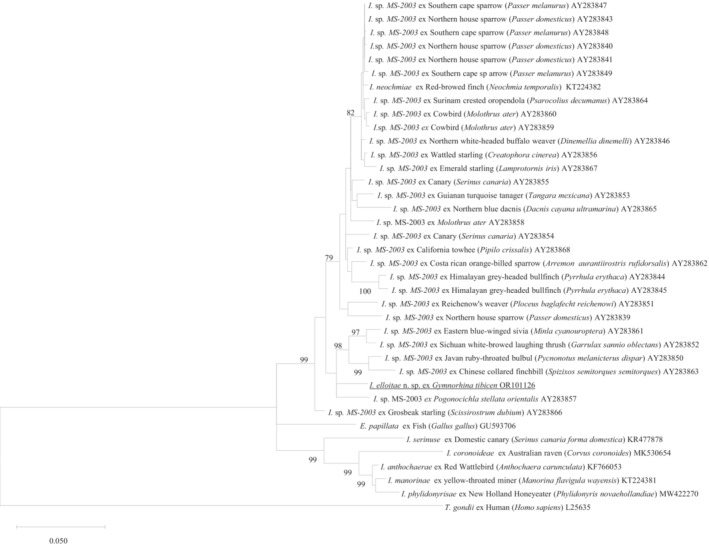
Evolutionary relationships of *Isospora elliotae* n. sp. inferred by distance analysis of 28S rRNA sequences. Percentage support (>70%) from 1000 pseudoreplicates from ML analysis.

A 1373 bp unique 28S rRNA sequence from *I*. *elliotae* n. sp. (*n* = 5) was compared with 35 *Isospora* spp. and one *Eimeria* sp. 28S sequences in GenBank (Figure 4), 29 of the *Isospora* 28S rRNA sequences were isolated from North American passerine birds characterized by Schrenzel et al. (2005) and six *Isospora* sequences from Western Australian passerine birds. It was shown that *I*. *elliotae* n. sp. sat in a large clade with an unnamed *Isospora* sp. (AY283857) along with the other 28 unnamed *Isospora* spp. from North America as well as *I*. *neochmiae* from Western Australia, which was separated by *Eimeria papillata* (GU593706) from the clade being composed of five Western Australian *Isospora* spp. (Figure 4).

Pair‐wise distance analysis revealed that *I*. *elliotae* n. sp. shared the highest genetic similarity at 97.2% with an unnamed *Isospora* sp. (AY283857) from a white‐starred robin (*Pogonocichla stellata* Vieillot, 1818) in North America.

*I*. *elliotae* n. sp. shared the highest genetic similarity of 96.2% with a Western Australia *Isospora* spp. from *I*. *neochmiae* (KT224382), from a captive‐bred red‐browed finch.

#### 3.2.3 COI gene

**FIGURE 5:**
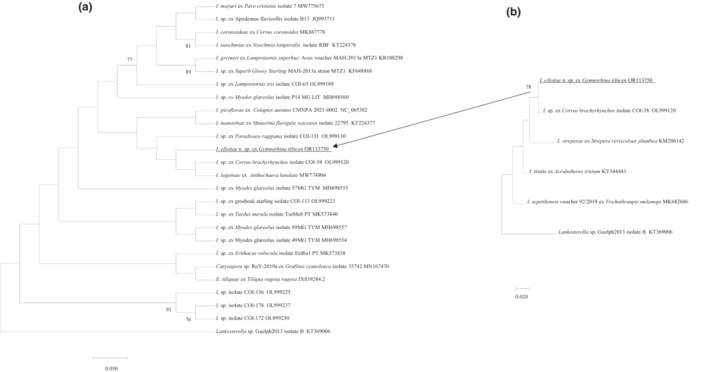
(a) Evolutionary relationships of *I. elliotae* n. sp. inferred by distance analysis of COI sequences. Percentage support (>70%) from 1000 pseudoreplicates from ML analysis based on a 633 bp. (b) Phylogenetic relationships of *I. elliotae* n. sp., *I. streperae*, *I. tristis*, *I. sepetibensis* and I. sp. ex *Corvus brachyrhynchos* isolate COI‐58 (231 bp COI only).

A 633 bp unique fragment of the COI gene of *I*. *elliotae* n. sp. (*n* = 3) was compared with 22 sequences from various *Isospora* sp., one *Eimeria* and one *Caryospora* sp. sequence. *Toxoplasma gondii* was used as outgroup (Figure 5). In the phylogenetic tree, *I*. *elliotae* n. sp. sat outside of the clade, which comprised *Isospora* spp. identified from passerine birds namely *I*. *lunulatae* (MW774904) isolated from a western wattlebird (*A*. *lunulata*) in Western Australia (Yang, Brice, Berto, & Zahedi, 2021) and the other was an unnamed *Isospora* sp. isolated from an American crow (*Corvus brachyrhynchos* Brehm, 1822) (OL999120) in America. The other *Isospora* spp., which grouped in the large clade with *I*. *elliotae* n. sp. on the COI tree, were all identified from North America (Figure 5a). Similarly, as with the 18S rRNA phylogenetic analysis, a sub‐tree including *I*. *streperae* was produced with 231 bp common COI sequences. *I*. *elliotae* n. sp. was in the same clade with an unnamed *Isospora* sp. isolate COI‐58 (OL999120), which was isolated from an American crow (Figure 5b).

The pair‐wise distance analysis showed *I*. *elliotae* n. sp. shared the highest genetic similarity of 99.2% with the unnamed *Isospora* sp. from an American crow (OL999120) and *I*. *lunulatae* (MW774904) from an western wattlebird Anthochaera in Western Australia and 98.2% with another unnamed *Isospora* sp. isolated from a raggiana bird of paradise (*Paradisaea raggiana* Sclater, 1873) (OL999130) in America.

## 4 DISCUSSION

Sporulated oocysts of *I*. *elliotae* n. sp. are morphologically different from other characterized *Isospora* spp. recorded from Artamidae or Passeriformes of Oceania (Madani et al., 2018; Trachta et al., 2006; Yang et al., 2014, Yang, Brice, Al Habsi, et al., 2015; Yang, Brice, Elliot, & Ryan, 2015, Yang, Brice, Jian, & Ryan, 2016, Yang, Brice, & Ryan, 2016, Yang, Brice, Berto, & Ryan, 2021, Yang, Brice, Berto, & Zahedi, 2021). As shown in Table 1, the oocyst dimensions of *I*. *elliotae* n. sp. (20.7 × 18.7 μm) are in a similar size range to those of *I*. *serini* (20.1 × 19.2 μm), which were identified from a canary (*Serinus canaria* Linnaeus) (Box, 1975; Table 1). The oocyst of *I*. *elliotae* n. sp. is subspherical in shape with a L/W ratio of 1.1 and a polar granule is present whilst the oocyst of *I*. *serini* is spherical in shape with a L/W ratio of 1.0 and has no polar granule. Another remarkable difference between *I*. *elliotae* n. sp. and *I*. *serini* is the sub‐Stieda body, which is significant in *I*. *serini* and indistinct in *I*. *elliotae* n. sp. Compared to *I*. *streperae*, which is the only described species that parasitizes the same host family Artamidae, its oocysts are larger, and it has a prominent sub‐Stieda body, whereas *I*. *elliotae* n. sp. has an indistinct or barely discernible sub‐Stieda.

**TABLE 1:** Morphological comparison of *Isospora elliotae* n. sp. with other *Isospora* species in the passerine birds.

*Coccidia*	Hosts	References	Oocysts						Sporocysts				
			Shape	Measurements (um)	Shape index	Wall (um)	Polar granule^a^	Oocyst residuum^a^	Shape^b^	Measurements	Stieda body	Substieda body	Residuum
*Isospora anthochaerae*	*Anthochaera carunculata*	Yang et al. (2014)	Subspherical	23.4 × 20.7 (20.0–26.0 × 19.0–22.0)	1.1	Bi‐layered c. 0.8	−	−	O	14.5 × 10.1 (11.0–17.0 × 9.0–11.0)	Hemi‐dome	Rectangular‐shaped	Compact
*Isospora braziliensis*	*Oryzoborus angolensis*	Trachta et al. (2006)	Spherical to subspherical	17.8 × 16.9 (16–19 × 16–18)	1	One‐layered c.1.0	−	−	E	13.2 × 10.8 (12–14 × 9–12)	Tiny	Absent	Scattered granules
*Isospora canaria*	*Serinus canaria Linnaeus*	Box (1975) and Berto et al. (2013)	Subspherical to ellipsoidal	24.6 × 21.8 (17–30 × 17–30)	1.1	Tri‐layered c. 1.2	+	−	Lemon	18.1 × 11.5 (17.0–22.0 × 1.00–13.0)	Nipple‐like	2.0 × 3.0	Compact
*Isospora curio*	*Oryzoborus angolensis*	Trachta et al. (2006)	Spherical to subspherical	24.6 × 23.6 (22–26 × 22–25)	1	Bilayerd c. 1.5	−	−	O	13.2 × 10.9 (15–17 × 10–13)	Small	Absent	Scattered granules
*Isospora daphnensis*	*Geospiza fortis*	McQuistion (1990)	Ellipsoidal	27.3 × 23.6 (22–30 × 20–27)	1.2	Bi‐layered c. 1.5	+	−	O	15.2 × 10.2 (15.0–16.0 × 9.0–11.0)	Nipple‐like	Small	Scattered granules
*Isospora elliotae* n. sp.	*Gymnorhina tibicen*	This study	Subspherical	20.7 × 18.7 (19.8–21.6 × 18–19.6)	1.1	Bi‐layered c. 1.5	+	−	O	12.6 × 9.7 (11.9–13.2 × 8.9–10.8)	Flattened to half‐moon	Indistinct	Compact
*Isospora exigua*	*Camarhynchus parvulus*	McQuistion and Wilson (1988)	Subspheroidal	20.4 × 20.1 (20–23 × 18–23)	1	One‐layered	−	−	O	14 × 9.5 (13–15 × 8–10)	Small	Small	Irregular‐shaped
*Isospora fragmenta*	*Camarhynchus parvulus*	McQuistion and Wilson (1988)	Subspheroidal	25.3 × 24.2 (24–27 × 23–25)	1	One‐layered	+	−	Piriform	15.4 × 11.5 (14–17 × 11–12)	Knob‐like	Prominent	Irregular‐shaped
*Isospora gryphoni*	Carduelis tristis *L*.	Olson et al. (1998)	Spherical	29.2 × 30.7 (25.0–33.0 × 28.0–34.0)	1	Bi‐layered c. 0.8	+	−	O	22.2 × 13.4 (15–25.0 × 12.0–14.5)	Small	Indistinct	Prominent
*Isospora lunulatae*	*Anthochaera lunulata*	Yang, Brice, Berto, and Zahedi (2021)	Subspheroidal	30.6 × 29.4 (27–34 × 26–31)	1.04	Bi‐layered c. 1.0	+	−	O	18.3 × 10.7 (17–19 × 10–12)	Flattened to rounded	Rounded to rectangular	Compact
*Isospora manorinae*	*Manorina flavigula obscura*	Yang, Brice, Jian, and Ryan (2016)	Spherical to subspherical	22.8 × 18.3 (20.3–23.8 × 17.7–18.7)	1.2	Bi‐layered c. 1.3	+	−	Lemon	15.5 × 9.7 (14.6–15.7 × 9.5–9.7)	Hemi‐dome	Rectangular‐shaped	Compact
*Isospora neochmiae*	*Neochmia temporalis*	Yang, Brice, and Ryan (2016)	Spherical	18.3 × 18.2 (18.2–18.9 × 18.2–18.6)	1	Bi‐layered c. 1.2	+	−	O	13.3 × 8.6 (9.5–16.4 × 6.8–10.0)	Indistinct	Absent	Compact
*Isospora paranaensis*	*Oryzoborus angolensis*	Trachta et al. (2006)	Subspherical to broadly ellipsoid	24.3 × 19.8 (22–26 × 18–22)	1.2	One‐layered c. 1.5	+	−	O	15.7 × 10.1 (14–18 × 8–12)	Distinct	Distinct	Spherical
*Isospora phylidonyrisae*	*Phylidonyris novaehollandiae*	Yang, Brice, Berto, and Zahedi (2021)	Subspheroidal	29.8 × 29.4 (29–32 × 28–31)	1.01	Bi‐layered c. 1.5	+	−	O	18.4 × 12.3 (18–19 × 12–14)	Flatted	Rounded	Scattered granules
*Isospora rotunda*	*Camarhynchus parvulus*	McQuistion and Wilson (1988)	Subspheroidal	20.9 × 20.8 (20–24 × 19–23)	1	One‐layered	+	−	O	15 × 9.7 (13–16 × 9–10)	Knob‐like	Prominent	Round
*Isospora serini*	*Serinus canaria* Linnaeus	Box (1975) and Speer and Duszynski (1975)	Spherical to subspherical	20.1 × 19.2 (13.0–23.0 × 13.0–23.0)	1	Tri‐layered c. 1.2	−	−	E	15.2 × 9.4 (13.0–16.0 × 8.0–11.0)	2.0 × 0.6	5.0 × 3.0	Scattered granules
*Isospora serinuse*	*Serinus canaria* forma *domestica*	Yang, Brice, Elliot, and Ryan (2015)	Spherical to subspherical	25.5 × 23.5 (24.4–27.0 × 22.0–24.8)	1.09	Bi‐layered c. 1.2	+	−	Lemon	18.9 × 11.8 (17.8–20.2 × 10.6–13.0)	Small	Indistinct	Compact
*Isospora streperae*	*Strepera versicolor*	Yang, Brice, Al Habsi et al. (2015)	Spherical	23.8 × 22.5 (22–24.5 × 21.8 × 24.5)	1.06	Bi‐layered c. 1.0	−	+	O	14.4 × 11.2 (11.5–15.8 × (10.4–12.5)	Hemi‐dome	Rectangular‐shaped	Compact
*Isospora temeraria*	*Geospiza fortis*	McQuistion and Wilson (1988)	Subspheroidal	25.4 × 21.1 (21–30 × 17–23)	1.2	One‐layered	+	−	Piriform	15 × 10 (14–15 × 9–11)	Knob‐like	Prominent	Round
*Isospora tristum*	Aves tristum	Madani et al. (2018)	Spherical to subspherical	23.3 × 22.3 (18.5–30 × 18.1–29.3)	1.05	Bi‐layered c. 1.3	−	−	O	13.9 × 9.3 (10.2–17.5 × 6.5–12.2)	Flatted	Rounded	Compact

^a^ −, absent; +, present.

^b^ E, ellipsoidal; O, ovoidal.

Molecular characterization of *I*. *elliotae* n. sp. at the 18S rRNA, 28S rRNA and COI loci showed that it did not match any of the sequences of *Isospora* spp. in the GenBank.

Due to funding constraints, only a few parasitological research groups worldwide conduct research on the taxonomy of coccidian parasites infecting wildlife. Due to most major disease research being carried out on those protozoan parasites infecting livestock and humans, there are only a limited number of publications available regarding *Isospora* taxonomy, both at the morphological and molecular levels.

Currently, another challenge facing *Isospora* taxonomy is that there is no international standard to refer to from the molecular aspect. How many targeted gene loci are required and which PCR primers are to be used to amplify the variable regions are some of the questions which need to be answered. An example of this can be seen in the study by Madani et al. (2018) when they characterized *I*. *tristum* in common mynahs (*Acridotheres tristis* Linnaeus, 1766). The authors obtained two short 18S rRNA sequences from the conserved region, one was 456 bp (KX216410) and the other was 439 bp (KX216411), which were not included in the phylogenetic analysis in our current study. When comparing these two sequences with the 18S rRNA from *I*. *elliotae* n. sp., the common region of the three sequences was identical. Further analysis of these two 18S rRNA from *I*. *tristum*, revealed that they were also identical to those from two *Isospora* spp. identified from *Myodes glareolus* (MH698574) (Trefancová et al., 2019) and the other isolated from *Passer melanurus* (AY331571) (Schrenzel et al., 2005). Therefore, the 18S sequence amplified from the conversed region in that study is not an ideal molecular maker to distinguish between *Isospora* spp. Fortunately, the authors also supplied a 304 bp COI gene sequence (KY344443), which shared 96.1% genetic similarity with that from *I*. *elliotae* n. sp. With reference to the morphological data discussed above, we are confident to claim that *I*. *elliotae* is a different species to that from *I*. *tristum*.

The *Isospora* spp., like other members of the Eimeriidae, are thought to be host‐specific and have co‐evolved with their definitive hosts (Biologie & Umweltwissenschaften, 1973; Kubiski et al., 2022; Power et al., 2009; Yang et al., 2012). Based on the extensive comparison of both the morphological and molecular data, *I*. *elliotae* n. sp. is a unique *Isospora* species from the Australian magpie (*Gymnorhina tibicen*).

Analysis of our genetic data from an *Isospora* origin and movement point of view, revealed that when the sequences at multiple genetic *loci* were examined, two groups of *Isospora* spp. were identified from birds in Western Australia. One group is closely related to the North America species/isolates (e.g. *I*. *neochmiae*; Yang, Brice, Jian, & Ryan, 2016) and *I*. *elliotae* n. sp.), and the other is a uniquely Australian group, which includes *I*. *anthochaerae* (Yang et al., 2014), *I*. *streperae* (Yang, Brice, Al Habsi, et al., 2015), *I*. *serinuse* (Yang, Brice, Elliot, et al., 2015), *I*. *manorinae* (Yang, Brice, Jian, & Ryan, 2016), *I*. *coronoideae* (Liu et al., 2020), *I*. *butcherae* (Yang et al., 2018) and *I*. *phylidonyrisae* (Yang, Brice, Berto, & Ryan, 2021).

## 5 CONCLUSION

This study provides morphological and genetic information on a novel *Isospora* species that infects the Australian magpie. The relationship between *Isospora* spp. from passerine birds from different continents, and their diversity, through morphological and molecular characterization to detail their relationships, deserves further study.

## AUTHOR CONTRIBUTIONS

**Rongchang Yang:** Conceptualization (lead); formal analysis (lead); methodology (lead); supervision (lead); writing – review and editing (lead). **Siobhon Egan:** Formal analysis (equal); methodology (equal); writing – review and editing (equal). **Huimin Gao:** Data curation (equal); methodology (supporting); writing – review and editing (equal). **Belinda Brice:** Investigation (equal); resources (equal); writing – original draft (equal). **Bruno P. Berto:** Data curation (equal); visualization (equal); writing – review and editing (equal).

## Data Availability

The 18S, 28S and COI additional sequence data generated from this study were accessible at the public domain: https://www.ncbi.nlm.nih.gov/nuccore under the GenBank accession numbers of OR101127, OR101126 and OR113750 for the 18S, 28S and COI loci, respectively after the paper is published. The 18S, 28S and COI additional sequence data used in the phylogenetic analysis were derived from the following resources available in the public domain: https://www.ncbi.nlm.nih.gov/nuccore with the GenBank accession numbers in the Figures 3, 4, 5.
